# A history of the shared airway: anaesthesia in ENT

**DOI:** 10.1017/S0022215125104246

**Published:** 2026-03

**Authors:** Alison Liu, John Adekoya

**Affiliations:** 1 King’s College London, London, UK; 2 University of Buckingham Medical School, Buckingham, UK

**Keywords:** anesthesia, otolaryngology, history of medicine, airway management, tonsillectomy

## Abstract

**Background:**

The specialties of ENT and anaesthesia have always had a unique relationship because of their longstanding history of co-operation over the shared airway.

**Methods:**

This historical review narrates how the modern practice of ENT surgery has developed following advances in anaesthetic techniques, as well as inspiring them.

**Results:**

From the earliest use of anaesthetic gases by Long, Wells and Morton, to their rapid adoption for use in tonsil and cleft palate surgical procedures, ENT surgeons were early beneficiaries of this new technology. The demands of surgery for facial injuries in World War II was a driver for anaesthetic advances, and Ivan Magill reinvented the specialty in response.

**Conclusion:**

Further developments in managing the shared airway, including jet ventilation, total intravenous anaesthesia and awake fibre-optic intubation, have shaped the modern ENT operating theatre, and highlight the vital collaboration between ENT and anaesthesia over the past 150 years.

## Introduction

The shared airway is a term used in otorhinolaryngology to describe the process of maintaining a patient’s patent airway whilst the surgeon operates in a similar anatomical space.^1^ ENT surgery has always had a unique relationship with anaesthesia because of the need for a shared airway. Developments in anaesthesia have enabled ENT surgeons to perform more complex operations, and the demands of ENT operations have also driven advances in anaesthetic technique. This narrative review covers highlights in the history of the shared airway.

### Historical anaesthesia

Many attempts have been made to reduce the excruciating pain of surgery. Until the arrival of modern anaesthesia, the best a patient could hope for was an intoxicant or sedative that would dull their senses while the surgeon operated as fast as they could. Eustachian tube catheterisation (1724), early attempts at mastoidectomy (1774) and myringotomy (1801) were all practiced in the age before reliable anaesthesia,^2^ when patients still relied on mixtures of opium, hemlock and alcohol to achieve insensibility.^3^

However, these potions were not always available, and neither were they reliably safe. Many operations were carried out without anaesthetic and therefore operative speed was of the essence. Tonsil guillotines were popular at this time as a way of performing tonsillectomy with maximum speed.

### The birth of modern anaesthesia

The 19th century saw the introduction of anaesthetic gases. The very first operations carried out using these novel anaesthetics were head and neck operations. Originally used for recreation in the form of pneumatic parties and ether frolics, their medical potential was realised when Crawford Long administered ether gas before removing a neck tumour from his patient John Venables in 1842. However, he did not publish an account of his operation until 1849, and it was William Morton who gave the first successful public demonstration of ether anaesthetic for the removal of a tumour from the jaw of Gilbert Abbot in 1846. William Morton and Charles Jackson filed a patent for the use of ether in a surgical procedure in November 1846, but the outrage from doctors and dentists at the idea of restricting this miraculous new technique was so great they quickly retracted their claim and made their methods freely available.^1^

### Anaesthetic gases in ENT surgery: from the sponge to the inhaler

The use of chloroform as an anaesthetic was first performed by Sir James Young Simpson in 1847, and both ether and chloroform were widely used in the following century. Inhaled anaesthesia in the UK was pioneered by John Snow in 1847, and he anaesthetised more than 148 children undergoing cleft lip repairs. Surgeons who had previously completed cleft lip repairs in as little as 20 seconds could develop a more aesthetic procedure.^4^ Cleft palate surgical procedures were initially seen as too risky to anaesthetise because of heavier bleeding and greater aspiration risk. However, Maurice Collis developed a cleft palate practice in Dublin using chloroform anaesthetic in 1865, and the use of anaesthetic gases for cleft operations became ubiquitous, allowing far more complex surgical techniques to be developed.^5^

Early methods for delivering anaesthetic gases were rudimentary, often involving sponges soaked in chloroform placed under the patient’s nose. This posed a challenge to surgeons operating in the head and neck area because they were at risk of inhaling a significant amount of anaesthetic fumes themselves. Furthermore, the difficulty in regulating anaesthetic dosage with sponges carried a high risk of overdose and fatalities. The invention of anaesthetic inhalers, such as Junker’s apparatus and Buxton’s bottle with safety valve, sought to address these issues by providing a more controlled and calculated delivery of anaesthetic gases. These innovations contributed to the broader acceptance and use of chloroform and ether in surgical procedures, enhancing the safety and effectiveness of anaesthesia and leading to improvements in surgical methods and outcomes with a reduced risk of injury to patients.^6^

As the use of inhaled anaesthesia became more widespread, it was rapidly incorporated into all aspects of head and neck surgery, and skilled anaesthetic administration became an indispensable part of surgical procedures. As highlighted in Berry and Legg’s operative manual, ‘the difference to the surgeon, between doing a cleft palate operation with a thoroughly experienced anaesthetist and an inexperienced one, is the difference between pleasure and pain!’^7^ Then, as now, surgeons were highly demanding of their anaesthetic colleagues.

### The first intubations: securing the airway for head and neck surgery

Whilst advances were made in the complexity of surgery as a result of anaesthetic gases, a significant challenge remained in controlling the airway and preventing aspiration when operating in proximity to the shared airway. This changed with the invention of endotracheal intubation. The late 19th century saw epidemics of diphtheria, and tracheotomy was widely practised as the treatment option of last resort in patients suffering from asphyxiation as a result of laryngeal membrane formation.^8^ However, survival rates were dire and there was a significant risk of complications from the procedure.^9^ Intubation techniques were developed by Eugene Bouchut in 1858 as a safer alternative.^10^

As intubation became more accepted as a medical technique, its potential benefits in head and neck surgical procedures became recognised. In 1871, Trendelenburg administered the first endotracheal anaesthetic and in 1878, Scottish surgeon Sir William Macewen performed the first recorded successful awake endotracheal intubation, to protect his patient’s airway prior to operating on an oral tumour.^11^

Intubation techniques made it possible to control the airway and prevent aspiration, and gave surgeons more room to operate in the head and neck area. Without endangering patient safety, intubation made it possible to develop increasingly sophisticated therapies, especially for surgical procedures performed around the shared airway. However, despite its potential, endotracheal intubation was not often used until the early 20th century, mostly because the necessary tools and methods were unavailable. This would all change with World War I.

### World War I: rectal ether anyone?

Airway management saw rapid progress because of the demands of World War I, as huge numbers of troops sustained facial and jaw injuries from shrapnel and high-velocity projectiles. Sir Harold Gillies completed more than 11 500 facial reconstructions at the Queen’s Hospital for Facial and Jaw Injuries in Sidcup. The severity of these facial injuries rendered standard chloroform inhalers ineffective for ensuring a secure airway and required surgeons and anaesthetists to share the space around the airway, necessitating the invention of new methods for delivering anaesthetic.

Captain Rubens Wade was one of Gillies’s first anaesthetists. In his attempt to improve operative access to the head and neck, he administered anaesthetic by rectal ether oil in more than 200 cases and routinely scrubbed to maintain the patient’s airway throughout surgery.^12^

Rectal ether vapour had first been used by Russian surgeon Ivan Pigorov in 1847, and the development of rectal ether oil in 1913 made it easier to administer. Rectal ether gave surgeons ample room to operate around the airway, minimised intra-operative interruptions to administer more anaesthetic gas and induction of anaesthesia was smoother with less coughing. However, it also required meticulous colonic preparation and there was a risk of overdose as it was difficult to remove the anaesthetic once instilled. Although several publications in the early 20th century recommended rectal ether for use in head and neck surgery, it never became widely adopted.^13^

The Sidcup anaesthetists also trialled endotracheal insufflation of anaesthetic gases to help manage the airway in Gillies’ operations. This was initially a very messy procedure, with the surgeon’s face and view obscured by vaporised anaesthetic and blood. However, Sir Ivan Magill and Stanley Rowbotham responded to this by passing a separate tube for the flow of expiratory gas, the so-called ‘Magill anaesthetic circuit’, a two-tube method of anaesthesia. Gillies could not have operated on so many complex patients without the support of Magill, who rewrote the field of anaesthesia during his time at Sidcup and whose inventions included the eponymous Magill laryngoscope, forceps and endotracheal tube.^14^ Despite the challenges faced, the advances made during this period allowed surgeons to operate safely for much longer lengths of time and on increasingly challenging cases.

### Operating in the airway: development of the laryngoscope

In the 20th century, ENT surgeons’ operative ambitions continued to expand. In pursuit of better access for working within the airway itself, operating laryngoscopes were developed from anaesthetic intubating blades. These new surgical laryngoscopes provided direct visualisation of the airway, a significant improvement from the indirect views obtained using mirrors. They also allowed instrumentation and operation further down the airway than ever before, giving surgeons the ability to make earlier diagnoses and perform safer treatments of a range of laryngeal diseases, such as tumours, vocal fold lesions and airway blockages.

American surgeon Chevalier Jackson was one such surgeon, and he developed new laryngoscopic equipment, including the eponymous Jackson laryngoscope. He used this equipment to visualise and remove at least 2347 inhaled and ingested foreign bodies, and he was so quick at removing objects from the airway and oesophagus that his assistants often remarked that he could remove foreign bodies faster than they could place them. He was famous for collecting all the objects he extracted, and in one memorable incident he refused to return a swallowed coin, even when its owner threatened to beat him for it.^15^

He was responsible for describing standardised protocols for foreign body removal and tracheotomy, and set up multiple educational courses to teach others how to replicate his techniques. This standardisation considerably improved the outcomes for endoscopic foreign body removal.^15^ His meticulous approach to airway management left a lasting impact on how ENT surgeons manage the airway.

### Boyle–Davis and Doughty: the modern tonsil gag

Despite advances in anaesthetic and intubation techniques, tonsillectomy was commonly performed with tonsil guillotine and ether induction alone well into the 1940s. Anaesthetic was rarely administered intra-operatively, and speed remained of the essence. Henry Boyle popularised the Boyle–Davis gag in 1921 and later incorporated a channel for ether gas administration, giving surgeons more time for dissection.^16^ However, the airway remained completely unsecured, and surgeons had to manage the shared airway intra-operatively to prevent aspiration of blood. In 1957, Doughty developed a slotted tongue blade that could hold an endotracheal tube securely in position, and intubation for tonsillectomies became more common.^17^

### Anaesthesia for the modern ENT operating theatre

Anaesthetic advances continue to be reflected in the modern-day ENT operating theatre.

#### Intensive Care Unit

In 1958, Dr Peter Safar founded the first intensive care unit in Baltimore. These units provided higher levels of peri-operative care, allowing ENT surgeons to take on more medically complex patients. They also gave surgeons greater confidence to operate around the airway because patients could be kept intubated with a secure airway post-operatively, reducing the risk of complications such as airway inflammation, collapse and obstruction.^18^

#### Jet ventilation

Ventilating bronchoscopes were developed in the 1950s but surgeons could not simultaneously instrument the airway and ventilate the patient. The manual jet ventilator was developed by Douglas Sanders in 1967. By passing oxygen at high pressure through a fine-bore adaptor attached to the rigid bronchoscope, the patient could be adequately ventilated through the Bernoulli effect whilst giving the surgeon unimpeded access to the airway.^19^

Further developments led to the creation of jet ventilation catheters that could be secured independently of a rigid bronchoscope, as well as high-frequency jet ventilation systems that automated jet delivery whilst reducing tidal volumes and therefore minimised vocal fold movement. Jet ventilation is today an essential technique to optimise the surgical access to the airway, particularly when the anatomy is narrow or distorted by pathology.^1^

#### Total intravenous anaesthesia

The development of total intravenous anaesthesia techniques with propofol in 1968 was also key to achieving tubeless anaesthesia. Total intravenous anaesthesia was advantageous because it offered a stable and uninterrupted anaesthetic without the need for anaesthetic gas delivery into the airway. It also provided hypotensive anaesthesia without tachycardia, minimising bleeding and improving haemostasis and visualisation of the surgical field. It is routinely used today for ENT procedures that require optimal access to the airway.^1^

#### Laser airway management

The use of laser surgery for laryngeal procedures has grown in popularity since the 1980s, but required specific anaesthetic adaptations to reduce the risk of airway fires and complete these procedures safely. Anaesthetists developed a wider range of equipment and ventilation strategies, including reinforced ‘laser-resistant’ endotracheal tubes, jet ventilation and tubeless field anaesthesia, to manage the airway in these cases.^20^ These strategies have enabled ENT surgeons to use the laser to operate in and around the airway with greater confidence.

#### Laryngeal mask airway

Laryngeal mask airways were invented by Archie Brain in the 1980s as an alternative to endotracheal tubes. They sit in the supraglottis and can be used to ventilate patients and administer volatile anaesthetic agents, as well as provide a conduit for endotracheal intubation. Prior to laryngeal mask airways, anaesthetists would have used a facemask and jaw thrust for the duration of short procedures such as myringotomy. Laryngeal mask airways freed up the hands of anaesthetists, reduced the risk of aspiration and gave surgeons better access to the face, nose and oropharynx.^21^

#### Intubating the difficult airway

For handling difficult airways, awake intubation, first described by Peter Murphy in 1967, has emerged as the ‘gold standard’. The fibre-optic scope made it possible to see the airway directly, as well as providing a manipulable guide for the endotracheal tube, making intubation safer and more successful in patients with difficult anatomy or other complicating conditions.^22^ This method was particularly helpful in ENT surgery because patients with head and neck cancers were more likely to have difficult intubations as a result of the disease or as a complication of treatment.

Additionally, intubation techniques have been substantially improved with the introduction of video laryngoscopy at the turn of the 21st century. Video laryngoscopes increased the success rate of intubations in patients with poor access to the airway and lowered the risk of complications by providing improved visualisation of the vocal folds and surrounding tissues.^23^ Video laryngoscopes and fibre-optic scopes also improved communication and teamwork by enabling numerous members of the surgical team to view high-definition images in real time and work together to manage patients with challenging airways.

Finally, trans-nasal humidified rapid insufflation ventilatory exchange was introduced from the respiratory ward and Intensive Care Unit to operating theatres in 2015, helping to maintain oxygen saturation and reduce carbon dioxide accumulation in apnoeic patients. It gave anaesthetists more time for intubation and significantly increased the margin of safety when securing difficult airways. It could also be used for pre-oxygenation, as an adjunct for sedation and to facilitate tubeless field surgery in ENT procedures.^24^

- There is a long history of collaboration between ENT and anaesthesia because of the nature of working around a shared airway, and advances in one field have consistently influenced the other
- The first demonstrations of inhaled anaesthetic gases were used in head and neck operations, and ENT surgeons were early beneficiaries of the new technique
- Sir Ian Magill played a pivotal role in developing the anaesthetic specialty during World War I, meeting the demands of facial reconstructive surgeon Sir Harold Gillies
- ENT surgeons, such as Chevalier Jackson, borrowed from anaesthetic equipment to develop their own operating laryngoscopes, allowing them to perform procedures further down the airway than ever before
- New technologies were developed from the 1960s onwards that are familiar in the modern ENT operating theatre, including jet ventilation, total intravenous anaesthetic and video laryngoscopes
- Collaboration between ENT and anaesthesia continues to drive innovation in managing complex cases and difficult airways today, and the historical partnership is stronger than ever

## Conclusion

Over the history of its development as a specialty, advances in ENT surgery have been driven by progress in anaesthetic techniques, and vice versa. Currently the two specialties continue to advance together. Surgeons are now able to push the limits of what is possible in operative ENT medicine thanks to innovations such as the creation of intensive care units, tubeless field anaesthesia and advanced airway management techniques for the difficult airway. Enhancements in patient care, safety and results are still being driven by the synergy between advancements in anaesthetic and surgical innovation. Future developments will enable ever more complex head and neck surgical procedures to be performed with greater safety and confidence. This collaborative approach is particularly significant in managing the challenges of the shared airway, where there is a long history of co-operation between the specialties of ENT surgery and anaesthesia.
